# Supplementary material for: COZOID: contact zone identifier for visual analysis of protein-protein interactions
Source: BMC Bioinformatics. 2018 Apr 6;19:125. doi: 10.1186/s12859-018-2113-6 (PMC5889581; doi:10.1186/s12859-018-2113-6)
Supplement: Supplementary file 2 — Software build. Executable binary file of the software tool. (ZIP 118,784 kb) [file 12859_2018_2113_MOESM2_ESM.zip › cozoid/platform/docs/testng-6.5.1-javadoc.zip!/org/testng/ClassMethodMap.html]

ClassMethodMap (TestNG)


---


|  |  |  |
| --- | --- | --- |
| **Overview** \| **Package** \| **Class** \| **Tree** \| **Deprecated** \| **Index** \| **Help** | |  |
| **PREV CLASS**   **NEXT CLASS** | **FRAMES**    **NO FRAMES**     **All Classes** |  |
| SUMMARY: NESTED \| FIELD \| CONSTR \| METHOD | DETAIL: FIELD \| CONSTR \| METHOD |  |


---


## org.testng Class ClassMethodMap

```
java.lang.Object
  org.testng.ClassMethodMap
```

---

``` public class ClassMethodMap extends java.lang.Object ```

This class maintains a map of `>`.
It is used by TestWorkers to determine if the method they just ran
is the last of its class, in which case it's time to invoke all the
afterClass methods.

**Author:**
:   Alex Popescu

---

**Constructor Summary**

`ClassMethodMap(java.util.List<ITestNGMethod> methods,
org.testng.internal.XmlMethodSelector xmlMethodSelector)`


| **Method Summary** | |
| --- | --- |
| `void` | `clear()` |
| `java.util.Map<ITestClass,java.util.Set<java.lang.Object>>` | `getInvokedAfterClassMethods()` |
| `java.util.Map<ITestClass,java.util.Set<java.lang.Object>>` | `getInvokedBeforeClassMethods()` |
| `boolean` | `removeAndCheckIfLast(ITestNGMethod m, java.lang.Object instance)`             Remove the method from this map and returns true if it is the last of its class. |

**Methods inherited from class java.lang.Object**

`clone, equals, finalize, getClass, hashCode, notify, notifyAll, toString, wait, wait, wait`

**Constructor Detail**

### ClassMethodMap

```
public ClassMethodMap(java.util.List<ITestNGMethod> methods,
                      org.testng.internal.XmlMethodSelector xmlMethodSelector)
```


**Method Detail**

### removeAndCheckIfLast

```
public boolean removeAndCheckIfLast(ITestNGMethod m,
                                    java.lang.Object instance)
```

:   Remove the method from this map and returns true if it is the last
    of its class.

---


### getInvokedBeforeClassMethods

```
public java.util.Map<ITestClass,java.util.Set<java.lang.Object>> getInvokedBeforeClassMethods()
```

---


### getInvokedAfterClassMethods

```
public java.util.Map<ITestClass,java.util.Set<java.lang.Object>> getInvokedAfterClassMethods()
```

---


### clear

```
public void clear()
```


---


|  |  |  |
| --- | --- | --- |
| **Overview** \| **Package** \| **Class** \| **Tree** \| **Deprecated** \| **Index** \| **Help** | |  |
| **PREV CLASS**   **NEXT CLASS** | **FRAMES**    **NO FRAMES**     **All Classes** |  |
| SUMMARY: NESTED \| FIELD \| CONSTR \| METHOD | DETAIL: FIELD \| CONSTR \| METHOD |  |


---
